# An ontological analysis of medical Bayesian indicators of performance

**DOI:** 10.1186/s13326-016-0099-4

**Published:** 2017-01-03

**Authors:** Adrien Barton, Jean-François Ethier, Régis Duvauferrier, Anita Burgun

**Affiliations:** 1 Département de médecine, Université de Sherbrooke, Sherbrooke, Québec Canada; 2 INSERM UMR 1099, LSTI, Rennes, France; 3 CHU de Martinique, Université Antilles-Guyane, Fort-de-France, France; 4 INSERM UMR_S 1138 Eq 22, Université Paris Descartes, Hôpital européen Georges Pompidou, AP-HP, Paris, France; 5 Centre de recherche du CHUS, CIUSSS de l’Estrie-CHUS, Sherbrooke, Québec Canada

**Keywords:** Sensitivity, Specificity, Medical test, Spectrum effect, Disposition, Realist ontology, Informational entity

## Abstract

**Background:**

Biomedical ontologies aim at providing the most exhaustive and rigorous representation of reality as described by biomedical sciences. A large part of medical reasoning deals with diagnosis and is essentially probabilistic. It would be an asset for biomedical ontologies to be able to support such a probabilistic reasoning and formalize Bayesian indicators of performance: sensitivity, specificity, positive predictive value and negative predictive value. In doing so, one has to consider that not only the positive and negative predictive values, but also sensitivity and specificity depend upon the group under consideration: this is the “spectrum effect”.

**Methods:**

The sensitivity value of an index test *IT* for a disease *M* in a group** g **is identified with the proportion of people in **g** who have *M* who would get a positive result to *IT* if the test *IT* was realized on them. This value can be estimated by selecting a reference test *RT* for *M* and a sample **s** of **g**, and measuring the proportion, among members of **s** having a positive result to *RT*, of those who got a positive result to *IT*. Similar approximation strategies hold for prevalence, specificity, PPV and NPV. Indicators of diagnostic performances and their estimations are formalized in the context of the OBO Foundry, built on the realist upper ontology Basic Formal Ontology (BFO).

**Results:**

Entities and relations from the Ontology for Biomedical investigations (OBI) and the Information Artifact Ontology (IAO) are used and complemented to represent reference tests and index tests, tests executions, tests results and the relations involving those entities, as well as the values of indicators of performance and their estimates. The computations taking as input several estimates of an indicator of performance to produce a finer estimate are also represented. The value of e.g. sensitivity estimates should be dissociated from the real sensitivity value – which involves possible, non-actual conditions, namely the result a person would get if a medical test would be performed on her. Such conditions could not be directly represented in a realist ontology, but a representation is proposed that introduces only actual entities by considering a disposition whose probability value is the real sensitivity value. A sensitivity estimate is a data item which is about such a disposition.

**Conclusions:**

This model provides theoretical basis for the representation of entities supporting Bayesian reasoning in ontologies.

## Background

### Definition of indicators of performance

Biomedical ontologies aim at providing the most exhaustive and rigorous representation of reality as described by biomedical sciences. A large part of medical reasoning deals with diagnosis and is essentially probabilistic. It would be an asset for biomedical ontologies to be able to support such a probabilistic reasoning.

Ledley and Lusted’s seminal article [1] on Bayesian reasoning in medicine defines different kinds of probabilistic entities. Consider for example the simple case of an instance of test of type *IT* (for “index test” – a test whose accuracy is being measured) aiming at detecting if a patient in a group **g** has an instance of disease of type *M*.^1^ The performance of test *IT* in diagnosing *M* can be quantified by the positive predictive value of this test, hereafter abbreviated PPV, defined by the *Oxford Handbook of Medical Statistics* [2] as the “proportion of tested positives who are true positives” and by the negative predictive value, hereafter abbreviated NPV, defined as the “proportion of tested negatives who are true negatives”. These values provide the probability that a patient has or not the disease, depending upon the result (positive or negative) to the test.

However, such values depend on some characteristics of the patient. If a patient received a positive test, the probability that he has the disease can for example depend upon his sex, his status of smoker or non-smoker, and other biological or environmental parameters. In particular, it depends on the prevalence of the disease among the group of persons with those characteristics.

Therefore, the statistical data communicated in the medical literature for a test are generally not the positive and negative predictive values, but the so-called “sensitivity” and “specificity”. The *Oxford Handbook of Medical Statistics* defines sensitivity as “the proportion of those who have the disease who are correctly identified by the test as positive” ([2], p. 340) and specificity as “the proportion of those who do not have the disease who are correctly identified by the test as negative“. The PPV and NPV can be computed on the basis of the prevalence Prev, sensitivity Se and specificity Sp thanks to the following Bayesian equations:

$$ PPV=\frac{Prev.Se}{Prev.Se+\left(1- Prev\right)\left(1-Sp\right)} $$

$$ NPV=\frac{\left(1- Prev\right).Sp}{Prev.\left(1-Se\right)+\left(1- Prev\right).Sp} $$

In the remainder of the article, sensitivity, specificity, PPV and NPV will be called “(Bayesian) indicators of performance” and abbreviated “IPs”.

In the wake of Ledley and Lusted [1] the sensitivity and specificity values have often been considered as depending only on the pathophysiological characteristics of the disease and of the test, and thus as being independent of the group of people under consideration. However, sensitivity and specificity values do in fact depend upon the group under consideration: this is the “spectrum effect” [3].

### The spectrum effect

If *IT* is an index test and *M* is a disease, let’s introduce f_1_(*IT,M*) as “the proportion of individuals who get a positive result to *IT*, among individuals who have *M*”, which fits with the usual definition of sensitivity (a﻿s provided by [2]). The main problem with this definition is that it does not specify the reference population. "The individuals who have *M*” are part of which population: the population in a given sample? The population of a specific country? The whole human population? Ledley and Lusted [1] considered that sensitivity and specificity depend upon pathophysiological characteristics of the disease, but not upon the population in consideration. If this was the case, the proportion of people tested positive among the diseased would be the same in any group under consideration – abstracting from statistical fluctuations due to randomness. However, as has been recognized by the medical literature, but regularly omitted, this hypothesis is false for at least two reasons. First, most tests are not inherently dichotomous but rely on a categorization of individuals based on continuous traits [3]. Second, various populations can express various disease characteristics (such as various degrees of severity [4]) that will influence the chance to get a positive result to a test.

**Fig. 1:**
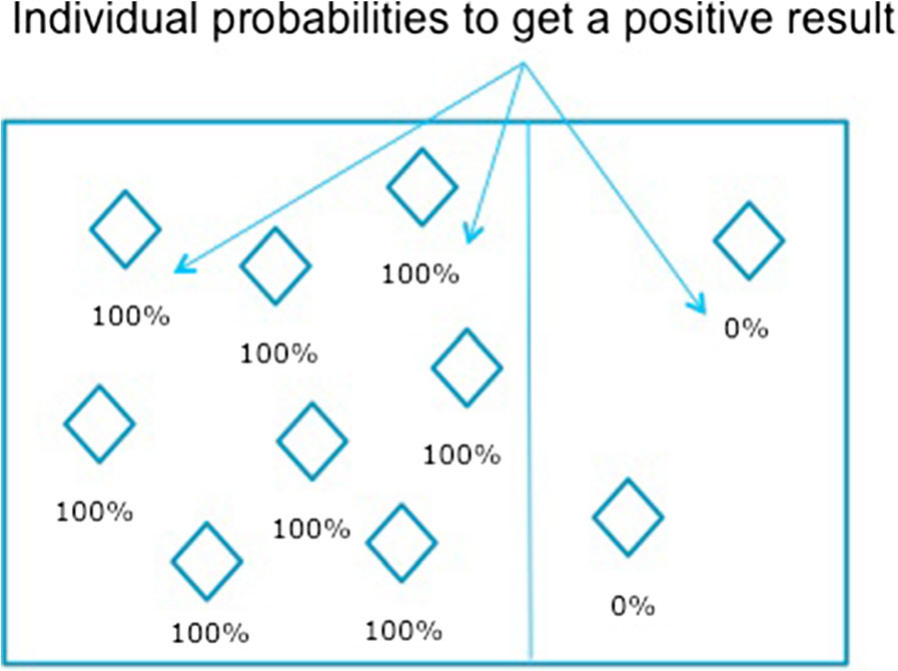
Variation of sensitivity depending on the group under consideration

The latter can be illustrated with the following example. Suppose that around 80 % of people having rheumatoid arthritis have a rheumatoid factor (RF), and would with certainty receive a positive result to a test that would perfectly^2^ detect this factor; and that the remaining 20 % do not have a rheumatoid factor, and would receive a negative result (yet do have the disease). The diseased population is then composed of two subgroups: a subgroup **sg**_**1**_ whose members would all get for sure a positive result to *IT*, and a subgroup **sg**_**2**_ whose members would all get for sure a negative result (see Fig. 1). The sensitivity calculated in this example would be 80 %.

Nevertheless, in reality, those proportions vary based upon various characteristics of the patients. For example, RF presence increases with age at onset of disease in juvenile arthritis [5]. As a result, the sensitivity of a test for RF will increase according to the age of the individuals of the population being tested. Its sensitivity will be lower in younger patients and higher in older patients.

Therefore, f_1_ is not a well-defined function: the value of the proportion does not depend only upon *IT* and *M*, but also upon the population **g** under consideration (which could be, for example, the whole human population, the Canadian smoker population, the female population, etc.). This is the “spectrum effect”, which can also be manifested, for example, as a dependence of sensitivity and specificity on the degree of severity of the disease in the group under consideration [4].

The sensitivity can therefore depend on the group **g** under consideration. A better candidate than f_1_(*IT,M*) to the definition of the sensitivity value would be the function f_2_(**g**,*IT,M*) defined as “the proportion^3^ among people in **g** who have *M* of those who would get a positive result to *IT if the test IT was realized on them*” – the mention in italic is necessary, as a test *IT* will not be realized on all individuals who have *M*, but on a sample only. The next part will distinguish three related entities: the real sensitivity^4^ value, its estimates, and the measurements of proportion in samples. It will also explain how such entities should be distinguished in an ontology of IPs.

## Methods

### Proportion measurement in a sample

It is impossible to know f_2_(**g**,*IT,M*) with certainty in practice﻿, for two reasons. The first reason is that it is often not possible to determine with certainty, through reasonable means, whether a given person has the disease *M* or not; in some cases, the only way to be certain would be to perform an autopsy on the deceased patient. Therefore, one needs to use a “reference test”, which is the best diagnostic test that is reasonable to perform in the present context (for more on the distinction between a reference test and the associated disease, see section “The challenge of representing indicators of performance in an ontology” below).

If the patient receives a positive result to this reference test, it will be concluded that he has the disease; if he receives a negative result, it will be concluded that he does not have it. But those inferences can be wrong: the reference test might lead to a positive result for a non-diseased person, or a negative result for a diseased person. If *RT* is a reference test for *M* and *IT* is an index test (of unknown accuracy) for *M*, then one can define the function f_3_(**g**,*IT,RT*) as “the proportion, among individuals of **g** who would get a positive result to *RT ﻿if﻿ th﻿e test RT had bee﻿n performed on them*, of people who would get a positive result to *IT if ﻿the﻿ test IT was realized on them*”. Since *RT* is a reference test for *M*, f_3_(**g**,*IT,RT*) approximates f_2_(**g**,*IT,M*). Both values can differ though: this is a first epistemic limit to the knowledge of f_2_(**g**,*IT,M*).

On top of this, f_3_(**g**,*IT,RT*) is not directly measurable. As a matter of fact, a test *IT* is never realized on a population as large as e.g., the whole population of smokers, or the whole male population. It is however possible to approximate f_3_(**g**,*IT,RT*) by performing both tests *IT* and *RT* on individuals in a sample **s** judged as being representative of the population **g**. Let’s define f_4_(**s**,*IT,RT*) as “the proportion, among members of **s** who got a positive result to *RT*, of those who got a positive result to *IT*”. If **s** is a representative sample of **g**, then f_4_(**s**,*IT,RT*) does approximate f_3_(**g**,*IT,RT*) – and thus, by transitivity, does approximate f_2_(**g**,*IT,M*). Note that as long as the sample **s** is not perfectly representative of **g**, f_4_(**s**,*IT,RT*) will differ at least slightly from f_3_(**g**,*IT,RT*) (which also differs from f_2_(**g**,*IT,M*)): this is a second limit to the knowledge of f_2_(**g**,*IT,M*).

Let’s illustrate those two limits of estimations with a study [4] which analyzes the quality of the Neer test (here written *IT’*) for diagnosing the shoulder impingement syndrome (written *M’*), a syndrome that is characterized by rotator cuff muscles inflammation near the sub-acromial space. In this study, the Neer test *IT’* is realized on a sample (written **s’**) of 552 patients, judged as representative of the target population (**g’**). Park et al. [4] take as reference test (*RT’*) the surgical observation. Here, f_4_(**s’**,*IT’*,*RT’*) is the proportion of people in the sample who have received a positive result to the Neer test, among those diagnosed as positive by surgical operation. f_4_(**s’**,*IT’*,*RT’*) approximates f_3_(**g’**,*IT’*,*RT’*), namely the proportion of individuals *in the target population* **g’** who would get a positive result to the Neer test among those who would get a positive result by surgical observation, if those tests were performed on them. Finally, f_3_(**g’**,*IT’*,*RT’*) itself approximates f_2_(**g’**,*IT’*,*M’*), which is the proportion of individuals in **g’** who would receive a positive Neer test result among those *who have an impingement syndrome*. Thus, f_4_(**s’**,*IT’*,*RT’*) approximates f_2_(**g’**,*IT’*,*M’*).

Note that similar approximation strategies hold for prevalence, specificity, PPV and NPV. Concerning e.g. specificity, one could thus define f’_2_(**g**,*IT,M*) as “the proportion^5^ among people in **g** who don’t have *M* of those who would get a negative result to *IT if the test IT was performed on them*”; and f’_4_(**s**,*IT,RT*) as “the proportion, among members of **s** who got a negative result to *RT*, of those who got a negative result to *IT*”. Thus, f’_4_(**s**,*IT*,*RT*) approximates f’_2_(**g**,*IT*,*M*).

### Sensitivity value and sensitivity estimates

Now that those definitions have been given, we can determine which entity the word ‘sensitivity’ refers to in the medical literature. At first sight, this term might appear polysemic. To illustrate this, let’s consider a study which evaluates the quality of an exercise test in the diagnosis of coronary artery disease, and claims: “The sensitivity varied substantially according to sex (women 30 % and men 64 %)” [6]. On one hand, the statement “sensitivity varies substantially according to the sex” suggests that sensitivity depends on the target population **g** in consideration, and that there is a sensitivity value for the female population, and another one for the male population. This formulation thus suggests that sensitivity value is given by the function f_2_(**g**,*IT,M*). However, the value 30 % assigned to the sensitivity of the test for women refers to a proportion which has been measured by the authors in a sample of 37 women, using coronary angiography as a reference test. This might thus suggest that the sensitivity value is in fact given by the function f_4_(**s**,*IT,RT*)

However, two arguments suggest that the sensitivity value should be interpreted as f_2_(**g**,*IT,M*) rather than f_4_(**s**,*IT,RT*). First, the value which is ultimately relevant for medical practice is f_2_(**g**,*IT,M*): if **s** is a sample of **g** and *RT* is a reference test for *M*, f_4_(**s**,*IT,RT*) is of interest for the medical practitioner only insofar as it provides an information on the disease *M* and the target population **g** from which the sample is taken – that is, insofar as it provides an estimate of f_2_(**g**,*IT,M*). Indeed, the fact that a few people who got a positive result to *RT* in a given sample have got a positive or negative result to a test *IT* has medical relevance only insofar as it teaches us something about how *diseased* people *in the target population* (not only in the sample) will react to this test *IT*.

Second, the sensitivity value is usually given with a 95 % confidence interval (see e.g., [7] or [8]), which estimates the likely range of error in determining the sensitivity value. But f_4_(**s**,*IT,RT*) can be measured with certainty,^6^ and thus the confidence interval cannot characterize the uncertainty on our knowledge of f_4_. On the other hand, there is some uncertainty on the knowledge of f_2_(**g**,*IT,M*) and f_3_(**g**,*IT*,*RT*), as they are estimated on the basis of f_4_(**s**,*IT,RT*). Therefore, the 95 % confidence interval would characterize the uncertainty on the knowledge of f_3_(**g**,*IT,RT*), which is taken as a proxy for f_2_(**g**,*IT,M*).^7^

Thus, those two arguments suggest that the term “sensitivity” should refer to f_2_(**g**,*IT,M*) – which is relative to a disease and a target population – rather than to f_4_(**s**,*IT,RT*) – which is relative to a reference test and a sample.^8^ As for f_4_(**s**,*IT,RT*), it can be interpreted as the value of a measurement of proportion in a sample, which provides an estimate of the sensitivity value.

Therefore, a sentence such as “The sensitivity varied substantially according to sex (women 30 % and men 64 %)” should, more rigorously, be formulated as: “The sensitivity varies substantially depending on the sex: through measurement of proportions in samples, its value was estimated to be 30 % for the women, and 64 % for the men”. We could prefer the first formulation, more compact, for practical reasons; but it is important to remember that it is only a shortcut for the second formulation.

Accordingly, we will need to dissociate three different kinds of entities. First, tests execution on a sample **s**, referring more precisely to the process of performing tests *IT* and *RT* and measuring the numbers of true positive, false positive, true negative and false negative as operationalized by *IT* and *RT* - for example, the false positive are people who are tested positive by the index test *IT* but negative by the reference test *RT* in the sample **s**. Second, the proportion of true positives among positives (as given by the reference test) is relative to the index test, the reference test and the sample, and its value is given by the function f_4_(**s**,*IT,RT*); as such, it provides an estimate of the sensitivity value. Third, the “real sensitivity”, which is relative to an index test, a disease and a population **g**, and whose value f_2_(**g**,*IT,M*) is given by the proportion of people in the group who would have a positive result to the test *IT* among those who are diseased. The real sensitivity would provide a better information than a sensitivity estimate on the probability that a random member of the group **g** would get a positive test result, in case he has the disease. However, its value f_2_(**g**,*IT,M*) cannot be known with certainty, contrarily to the value of the sensitivity estimate f_4_(**s**,*IT,RT*).

More generally, those considerations can be adapted to other indicators of performance (specificity, PPV and NPV), as well as the prevalence. In particular, f’_2_(**g**,*IT,M*) should refer to the real specificity value, whereas f’_4_(**s**,*IT,RT*) can be interpreted as the value of a measured proportion in a sample that provides an estimate of the real specificity value. In particular, *real* sensitivity, specificity, PPV and NPV, as we have defined them above, depend neither on the sample nor on the reference test. However, they are estimated on the basis of proportion *measurements* which depend both on the sample and the reference test. Accordingly, when a study [9] mentions “cadaveric prevalence” of the rotator cuff tears, this expression should be understood as a linguistical shortcut denoting a proportion measurement in a sample when the cadaverical analysis is adopted as reference test; and the “radiological prevalence” should be understood as a proportion measurement when the radiological analysis is adopted as reference test. The real prevalence, however, does not depend on the reference test.

### Aggregation of sensitivity estimates

Finally, we need to add a last layer to this model. Approximations of sensitivity taken in different samples, with different index tests, can be combined in order to build a finer estimate of sensitivity for a more encompassing category of index tests. Consider for example the meta-analysis [7] which assess the quality of peripheral thermometers in detecting fever. They use as reference test a pulmonary artery catheter, and consider 29 studies assessing the sensitivity and specificity of those devices. Combining those values, they come up with an estimate of 0.64 for the sensitivity and of 0.96 for the specificity.

### The challenge of representing indicators of performance in an ontology

To the extent that they aim at representing biomedical knowledge and enabling medical reasoning, biomedical ontologies should provide a formalization of IPs as well as the prevalence, by dissociating e.g. the real sensitivity from the sensitivity estimates, and the process leading to those estimates. This article will introduce such a formalization in the context of the OBO Foundry [10], one of the most massive set of interoperable ontologies in the biomedical domain, built on the upper ontology Basic Formal Ontology (BFO) 1.1 [11].

BFO endorses a realist methodology, which carefully dissociates material entities (such as disorders) from informational entities (such as diagnosis). In common medical practice, a disease may be diagnosed in ideal circumstances by a given gold standard test, which can be defined as the most accurate reference test; but the disease, the diagnosis, and the result to a gold standard test are three different entities that should be distinguished. As a matter of fact, many human diseases already existed a few thousands of years ago, much before they could be diagnosed. Moreover, a diagnosis can be wrong or imprecise. Finally, a given gold standard can be later replaced by a better one: this shows that the disease cannot be defined by a positive result to a gold standard - otherwise, there could not be, by definition, a “better” gold standard. Thus, while a diagnosis of a disease represents the best knowledge by some health or research professional of the presence of the disease in a particular patient, a diagnosis is not equivalent to a disease: it is rather “about” a disease. This formalization is compatible with IAO (Information Artifact Ontology [16]) and OGMS (Ontology for General Medical Sciences).

The question of how probabilistic notions can be represented in ontologies has been tackled from different perspectives in the past. For example, [12] has proposed the alternative PR-OWL format that extends the classical OWL format; we take here a different approach, which does not aim at changing the OWL format. Soldatova and colleagues [13] have described a model in which probabilities can be assigned to research statements. We build here upon an alternative approach [14], in which probabilities can be assigned to dispositions.

Sensitivity and specificity have been recently introduced in the Ontology of Biological and Clinical Statistics (OBCS [15]) as subclasses of *Data item*. We will partly endorse and refine this classification, by considering estimates of sensitivity and specificity as subclasses of *Data Item*, and extend this classification to PPV and NPV. A data item, as defined by the Information Artifact Ontology (IAO) [16], is intended to be a truthful statement about something. In order to formalize IPs, one should thus clarify which entities in the real world they are about.

Proportion measurements are data items that are obtained from some processes named "proportion measures", which involve performing two kinds of tests (the index test and the reference test) in a sample. On the other hand, we have defined a real sensitivity value f_2_(**g**,*IT,M*) as the proportion of people who would get a positive result by *IT* among those who have the disease *M.* But note here the conditional structure: what is referred to is the proportion of true positives among diseased *if IT* was performed on them. In realistic situations, however, as explained above, the sensitivity value will be estimated by performing the test on a sample of the population only – not the entire population **g**; thus, f_2_(**g**,*IT,M*) is the value of a non-actual proportion.^9^ However, possible-but-non-actual situations cannot be straightforwardly represented in a realist ontology like BFO. To solve this problem, we will formalize the real IP value as the probability assigned to a disposition borne by an instance of group of individuals; and estimates of IPs as data items which are about such a disposition. This will provide a formal characterization of IPs and their estimates based on proportion measurements.

## Results

**Fig. 2:**
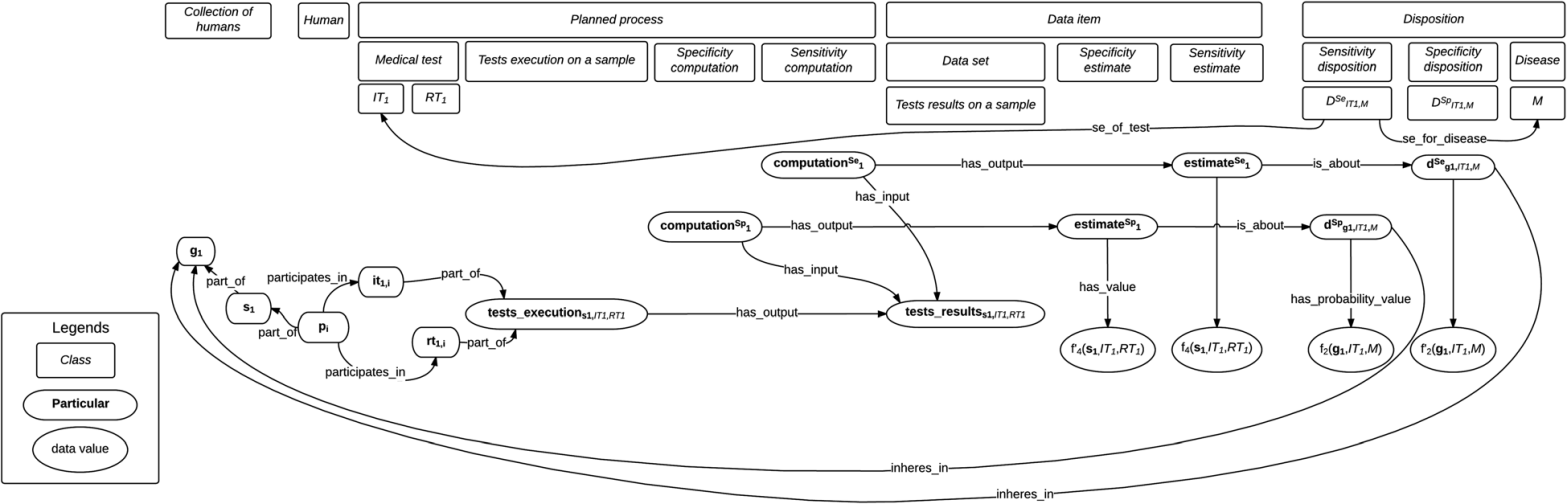
Real sensitivity and specificity values and their estimates

**Fig. 3:**
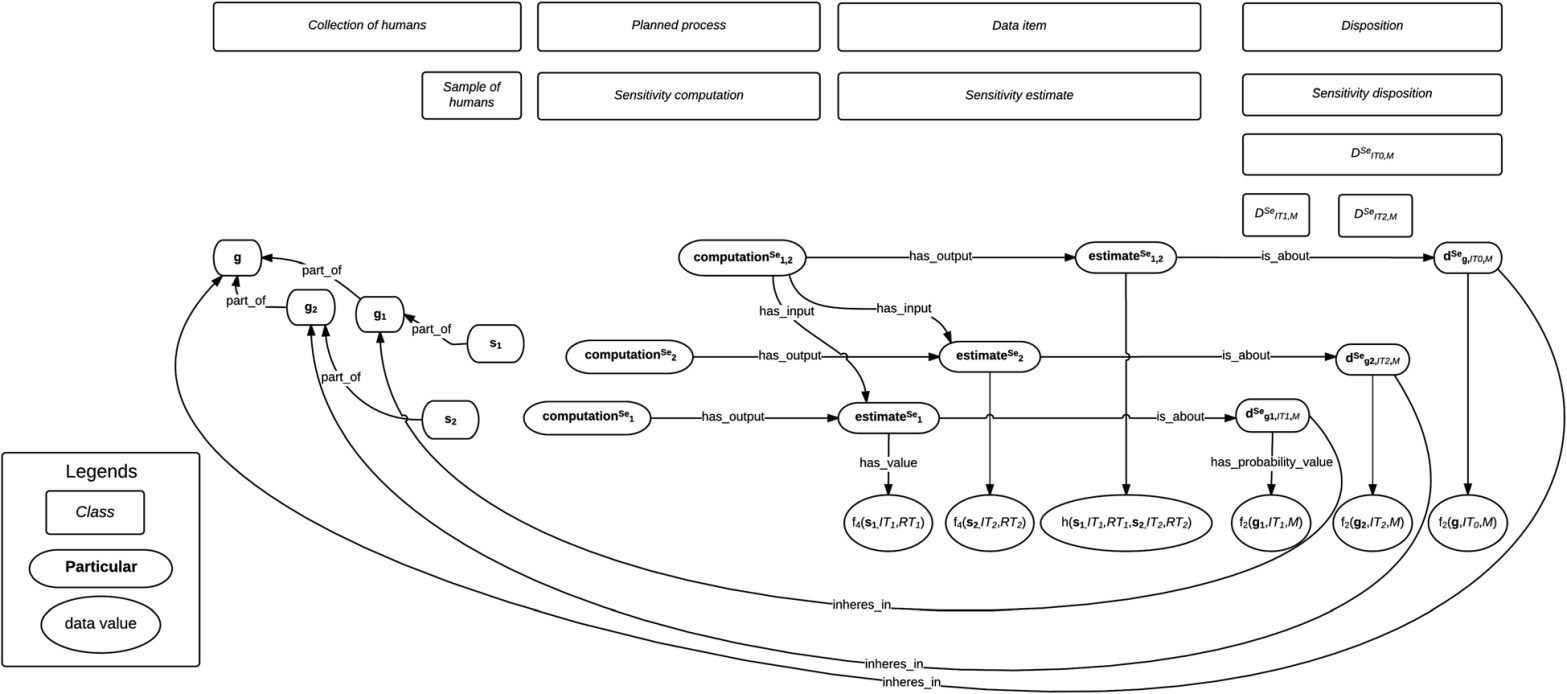
Aggregation of several sensitivity estimates

The formalization that will be presented here can be visualized on Fig. 2 and Fig. 3, in which classes are in rectangles, instances in boxes with rounded edges, and the numerical value assigned by datatype properties in ellipses. Unless specified otherwise, all the relations used here belong to BFO 1.1 [11].

### Test results and sensitivity estimate

Let us first start with the formalization of test results and the IP estimates they lead to (see Fig. 1).^10^ A *Medical_test* will be here considered as a subclass of *Planned_process* (as defined by OBI, the Ontology for Biomedical Investigations [17]) which consists in the observation of a given feature to infer the presence of another feature – in the case of interest, a pathological entity such as a disease. Consider a medical test^11^ *IT*_*1*_ and a disease *M*:

- *Medical_test is_a Planned_process*
- *IT*_*1*_ *is_a Medical_test*
- *M is_a Disease*

Suppose that we are interested in the sensitivity and specificity of test *IT*_*1*_ for diagnosing *M* in a group **g**_**1**_. This group **g**_**1**_ will be formalized as a collection of humans (for more on collections, see [18]). To estimate this sensitivity and specificity, one can select a sample **s**_**1**_ considered to be representative of **g**_**1**_ (which will be called the reference class). Thus:

- **g**_**1**_ **instance_of** *Collection_of_humans*
- **s**_**1**_ **instance_of** *Sample_of_humans*
- *Sample_of_humans is_a Collection_of_humans*
- **s**_**1**_ **part_of g**_**1**_

Let’s now introduce the class of tests *RT*_*1*_ which are reference tests for *M*:

- *RT*_*1*_ *is_a Medical_test*

**s**_**1**_ is composed of *n* humans, named **p**_**1**_, **p**_**2**_,…,**p**_**n**_. Two^12^ tests will be performed on each **p**_**i**_: an instance of *RT*_*1*_, named thereafter **rt**_**1,i**_, and an instance of *IT*_*1*_, named **it**_**1,i**_; thus, for every *i* between 1 and *n*:

- **p**_**i**_ **instance_of** *Human*
- **p**_**i**_ **part_of s**_**1**_
- **p**_**i**_ **participates_in rt**_**1,i**_
- **p**_**i**_ **participates_in it**_**1,i**_

We introduce **tests_execution**_**s1,***IT1,RT1*_ which has as part all the tests **rt**_**1,i**_ and **it**_**1,i**_ for i between 1 and n and the recording of which members of the sample are true positives (those who have been tested positive both by *IT*_*1*_ and *RT*_*1*_), true negatives (those who have been tested negative both by *IT*_*1*_ and *RT*_*1*_), false positives (those who have been tested positive by *IT*_*1*_ but negative by *RT*_*1*_) and false negatives (those who have been tested negative by *IT*_*1*_ but positive by *RT*_*1*_). This recording leads (OBI:**has_specified_output**) to the creation of the instance of *Data_set* named **tests_results**_**s1,***IT1,RT1*_:

- **tests_execution**_**s1,***IT1,RT1*_ **instance_of** *Planned_process*
- **rt**_**1,i**_ **part_of tests_execution**_**s1,***IT1,RT1*_
- **it**_**1,i**_ **part_of tests_execution**_**s1,***IT1,RT1*_
- **tests_results**_**s1,***IT1,RT1*_ **instance_of** *Data_set*
- **tests_execution**_**s1,***IT1,RT1*_ **has_specified_output tests_results**_**s1,***IT1,RT1*_

The **tests_results**_**s1**,*IT1,RT1*_ will then serve as input (OBI:**has_specified_input**) to a planned process noted **computation** ^**Se**^_**1**_ which computes a sensitivity estimates noted **estimate** ^**Se**^_**1**_, by calculating the proportion of true positives among positives:^13^

- **computation** ^**Se**^_**1**_ **is_a** *Planned_process*
- **estimate** ^**Se**^_**1**_ **is_a** *Data_item*
- **computation** ^**Se**^_**1**_ **has_specified_input tests_results**_**s1,***IT1,RT1*_
- **computation** ^**Se**^_**1**_ **has_specified_output estimate** ^**Se**^_**1**_

Finally, we can use the datatype property OBI:**has_specified_value** to relate **estimate** ^**Se**^_**1**_ with its numerical value f_4_(**s**_**1**_,*IT*_*1*_,RT_*1*_):

- **estimate** ^**Se**^_**1**_ **has_specified_value** f_4_(**s**_**1**_,*IT*_*1*_ *,RT*_*1*_)

Similar strategies can hold for representing Specificity, PPV and NPV and their estimates.^14^

### Aggregation of sensitivity estimates

We will now show how various sensitivity estimates can be aggregated for a finer sensitivity estimate (cf. Fig. 3). Suppose that we have another sample **s**_**2**_ (also a **part_of g**), composed of n’ humans named **q**_**1**_,** q**_**2**_, ..., **q**_**n'**_. We can perform another measure of sensitivity for a related (possibly identical to *IT*_*1*_) index test *IT*_*2*_ for *M* in **g** on this sample, using a related (possibly identical to *RT*_*1*_) reference test *RT*_*2*_, by performing instances of *RT*_*2*_ named **rt**_**2**,j_ (for j between 1 and n’) and instances of *IT*_*2*_ named **it**_**2,j**_ on each member **q**_**j**_ of **s**_**2**_. One can then define the entity **tests_execution**_**s2,***IT2,RT2*_ as a planned process which has as part those tests **rt**_**2**,j_ and **it**_**2,j**_, and which has as output **tests_results**_**s2,***IT2,RT2*_; the latter serves as input to another computation of sensitivity **computation** ^**Se**^_**2**_, which has as output another estimate of sensitivity **estimate** ^**Se**^_**2**_, to which the value f_4_(**s**_**2**_,*IT*_*2*_ *,RT*_*2*_) can be assigned (the latter being the proportion, among people who have been tested positive by *RT*_*2*_ in **s**_**2**_, of people who had a positive result to *IT*_*2*_).

As explained earlier, various sensitivity estimates can be combined to estimate the value of the sensitivity of a test for *M* in **g**. If *IT*_*1*_ and *IT*_*2*_ on one hand, and *RT*_*1*_ and *RT*_*2*_ on the other hand, are similar enough (in particular, if they are identical), those results might be gathered to come up with a finer estimate of the sensitivity value. More specifically, if *IT*_*1*_ and *IT*_*2*_ can be subsumed under a common index test class *IT*_*0*_, and *RT*_*1*_ and *RT*_2_ can also be subsumed under a common reference test class *RT*_*0*_, then their values can be compiled mathematically (for example by meta-analysis methods) to come up with the value of a (hopefully finer) estimate named **estimate** ^**Se**^_**1,2**_, whose value is given by a function h(**s**_**1**_,*IT*_*1*_,*RT*_*1*_,**s**_**2**_,*IT*_*2*_,*RT*_*2*_). This can be generalized to the aggregation of more than two former estimates.

We can here introduce a planned process of computation of sensitivity named **computation** ^**Se**^_**1,2**_, which takes as input both **estimate** ^**Se**^_**1**_ and **estimate** ^**Se**^_**2**_, and the output of such a process, a data item named **estimate** ^**Se**^_**1,2**_:

- **computation** ^**Se**^_**1,2**_ **instance_of** *Planned_process*
- **estimate** ^**Se**^_**1,2**_ **instance_of** *Data_item*
- **computation** ^**Se**^_**1,2**_ **has_specified_input estimate** ^**Se**^_**1**_
- **computation** ^**Se**^_**1,2**_ **has_specified_input estimate** ^**Se**^_**2**_
- **computation** ^**Se**^_**1,2**_ **has_specified_output estimate** ^**Se**^_**1,2**_
- **estimate** ^**Se**^_**1,2**_ **has_specified_value** h(**s**_**1**_,*IT*_*1*_ *,RT*_*1*_ *,* **s**_**2**_,*IT*_*2*_ *,RT*_*2*_)

We will not aim at giving the details of this function h, which is the responsibility of the statistician, not the ontologist – who focuses on how to represent such values.

Finally, since **estimate** ^**Se**^_**1**_ or **estimate** ^**Se**^_**1,2**_ are informational entities, they must be about some entities. To determine what those entities are about, we will need to formalize the entity to which is assigned the “real sensitivity value”.

### Real sensitivity value

As said earlier, estimates of sensitivity of *IT* for *M* in **g** aim at estimating the real sensitivity value, which is given by the proportion of members of **g** who would get a positive result to *IT* among those who have *M*. However, the condition of performing the test *IT* on the members of **g** is never realized, because the test is performed (at best) on one or several samples of the population, not on the whole population **g**: the performance of test *IT* on the members of **g** is a *possible* (leaving aside practical difficulties), *non-actual* condition. Interpreting specificity, PPV, and NPV along the former lines would also imply such possible, non-actual conditions.

BFO’s realist methodology [19] implies that all instances should be *actual* entities. Thus, one cannot represent directly such a possible-but-not-actual condition in an ontology based on BFO. In order to solve this difficulty, we will introduce a strategy named “randomization”, which will clarify the nature of the real sensitivity value as a probability assigned to an actual entity, namely a disposition. This will also clarify what an estimate of sensitivity is about, namely about this disposition. Thus, it will enable to represent IPs in a realist fashion, compliant with BFO’s methodology.

### From proportions to objective probabilities: the randomization strategy

We will explain first how the proportion of a subgroup in a group can be formalized as a probability value assigned to a disposition; this will help explaining later how the proportion of a subgroup in a group undergoing a possible, non-actual condition can be formalized along similar lines.

Dispositions are entities that can exist without being manifested; an example of disposition is the fragility of a glass, which can exist even when the glass does not break. We will use Röhl & Jansen's model of disposition [20] in BFO, which associates to every instance of disposition one or several instances of realizations, and one or several instances of triggers (a trigger is the specific process that can lead to a realization occurring). In this model, the fragility of a glass is a disposition of the glass to break (the breaking process is the realization) when it undergoes some kind of stress (the process of undergoing such a stress is the trigger); this disposition inheres in the glass. Starting with the definition of these entities and their relations at the instance level, Röhl & Jansen proceed to formalize them at the universal level. Previous work [14] has shown how to adapt this model to probabilistic dispositions. Thus, an instance of balanced coin is the bearer of an instance of disposition to fall on heads (the realization process) when it is tossed (the trigger process), to which an objective probability 1/2 can be assigned.

We will now extend the scope of this model to the situation at hand. Consider the prevalence Prev(**g**,*M*), which was defined above as the proportion of persons having *M* in the actual population **g**. We can define the disposition **d** ^Prev^_**g**,*M*_, borne by the group **g**, that a person randomly drawn in **g** has *M*. More specifically, let’s write *T*_**g**_ the process “randomly drawing a person in **g**”, and *R*_***g****,M*_ the process “drawing by *T*_**g**_ someone who has *M*”: the triggers of **d** ^Prev^_**g**,*M*_ are instances of *T*_**g**_ and its realizations are instances of *R*_**g***,M*_. Following the lines of previous work [14], one can thus define the probability assigned to the disposition^15^ **d** ^Prev^_**g**,*M*_, which is the probability of drawing randomly someone who has *M* in **g**. This probability is equal to the proportion of individuals who have *M* in **g**, that is, to Prev(**g**,*M*): if there are e.g., 10 % diseased people in **g**, then the probability of drawing randomly a diseased person in **g** is 10 %. Thus, the prevalence value can be identified to the objective probability assigned to the disposition **d** ^Prev^_**g**,*M*_. We name this strategy the “randomization” of the proportion of persons having *M* in **g**.

The randomization strategy may not be necessary to formalize a proportion in an actual group, such as the prevalence. But this strategy can also be applied to proportions of people in groups which are subject to a *possible, non-actual* condition – and thus, be relevant to formalize sensitivity and other IPs, and their estimates. As a matter of fact, the real sensitivity value f_2_(**g**,*IT,M*) was defined as the proportion of people who would get a positive result to *IT* among *M*’s bearers in **g**. This value can be “randomized” as follows. We can define **d** ^Se^_**g**,*IT,M*_ as the disposition^16^ to draw randomly, among the individuals of ***g*** who have *M*, someone who is tested positive by *IT*. More specifically, let’s define the process *T* ^Se^_**g***,IT,M*_ as the “performance of test *IT* on the individuals in **g**, and random draw of an individual among those who have the disease *M*”;^17^ and the process *R* ^Se^_**g***,IT,M*_ as the “drawing by *T* ^Se^_**g***,IT,M*_ of someone who got a positive result to *IT*”. The triggers of **d** ^Se^_**g**,*IT,M*_ are instances of *T* ^Se^_**g***,IT,M*_, and its realizations are instances of *R* ^Se^_**g***,IT,M*_ . As it happens, the real sensitivity value f_2_(**g**,*IT,M*) is the objective probability assigned to this disposition **d** ^Se^_**g**,*IT,M*,_: indeed, if there are e.g., 15 % of the diseased people in **g** who would get a positive result by *IT*, then the probability of randomly drawing someone who got a positive test result by *IT* among diseased people in **g** if test *IT* would be performe﻿d﻿ o﻿n them﻿ is equal to 15 %.

Specificity value can be defined along similar lines, as probabilities assigned to actual dispositions borne by the group g noted **d** ^Sp^_**g**,*IT,M*_ (and similarly for the PPV and NPV). Although both **d** ^Se^_**g**,*IT,M*_ and **d** ^Sp^_**g**,*IT,M*_ are dispositions inhering in **g**, they have different triggers and different realizations; the process *T* ^Sp^_**g***,IT,M*_ is the “performance of test *IT* on the individuals in **g**, and random draw of an individual among those who *do not* have the disease *M*” and the process *R* ^Sp^_**g***,IT,M*_ is the “drawing by *T* ^Sp^_**g***,IT,M*_ of someone who got a *negative* result to *IT*”.

### Assignment of real sensitivity values to dispositions

Let us now consider how to formalize these probability values in ontologies. **d** ^Se^_**g**,*IT,M*_ is a disposition individual inhering in the group **g**; and a probability value can be assigned to this disposition using a datatype property **has_probability_value** [15]. This probability value is what we called the real sensitivity value:^18^

- **d** ^Se^_**g**,*IT,M*_ **has_probability_value** f_2_(**g**,*IT,M*)

Thanks to our analysis above, we can now answer our original question, and state what sensitivity estimates such as **estimate** ^**Se**^_**1**_ or **estimate** ^**Se**^_**2**_ are about^19^ - namely, about this disposition:

- **estimate** ^**Se**^_**1**_ **is_about d** ^Se^_**g1**,*IT1,M*_
- **estimate** ^**Se**^_**2**_ **is_about d** ^Se^_**g2**,*IT2,M*_

Also, if the samples **s**_**1**_ and **s**_**2**_ are considered by the statistician as representative enough of a general population **g**_**0**_ encompassing **g**_**1**_ and **g**_**2**_, if *RT*_*1*_ and *RT*_*2*_ are considered as similar enough to be representative in the same way of the disease *M*, and if *IT*_*1*_ and *IT*_*2*_ are considered as similar enough to be representative of a more general index test *IT*_*0*_, then:

- **estimate** ^**Se**^_**1,2**_ **is_about d** ^Se^_**g0**,*IT0,M*_

As **d** ^Se^_**g**,*IT,M*_ is an individual, it cannot be related directly to the classes *IT* and *M*, but only indirectly, through the following formalization. First, **d** ^Se^_**g**,*IT,M*_ can be seen as an instance of a disposition class written *D* ^*Se*^_*IT,M*_, which has as trigger the process class *T* ^Se^_*IT,M*_: “performance of test *IT* on the members of a group, and random draw of a person among those who have the disease *M*”; and as realization the process class *R* ^Se^_*IT,M*_ defined as “drawing by *T* ^Se^_*IT,M*_ of someone who got a positive result to *IT*”. We can then introduce two new relations *sensitivity_disposition_of_test* and *sensitivity_disposition_for* (abreviated as *se_of_test* and *se_for_disease*) relating *D* ^Se^_*IT,M*_ with *IT* and *M*:

- **d** ^Se^_**g**,*IT,M*_ **instance_of** *D* ^Se^_*IT,M*_
- *D* ^Se^_*IT,M*_ *is_a Disposition*
- *D* ^Se^_*IT,M*_ *se_of_test IT*
- *D* ^Se^_*IT,M*_ *se_for_disease M*

These two relations *se_of_test* and *se_for_disease* are introduced for pragmatic reasons of facility of use: on a foundational level, *D* ^*Se*^_*IT,M*_ and *M* (resp. *IT*) could be related through a complex array of relations and entities that involve the relation *has_trigger* between *D* ^*Se*^_*IT,M*_ and *T* ^*Se*^_*IT,M*_, as well as a sequence of relations between *T* ^Se^_*IT,M*_ and *M* (resp. *IT*). Such an analysis would raise interesting theoretical questions, as instances of *D* ^*Se*^_*IT,M*_ can exist even if no instance of *M* or *IT* do exist - we therefore face here issues similar to the ones addressed by [20] and [21].

Figure 2 represents classes and particulars involved in formalizing tests execution and results, sensitivity estimates, the disposition this estimate is about, and the real sensitivity value. Figure 3 represents the classes and particulars involved in formalizing aggregation of sensitivity estimates into a finer estimate. Specificity, PPV and NPV can be formalized along similar lines, as data items about dispositions related to tests and diseases through relations that could be labeled *sp_of_test*, *sp_for_disease*, *ppv_of_test, ppv_for_disease*, *npv_of_test,* and *npv_for_disease.*

### Example of application

An example will now illustrate this formalization. McTaggart and colleagues [8] have performed a meta-analysis to determine the accuracy of point-of-care tests for detecting albuminuria (let’s call *IT*_*0*_ the class of such index tests), using as reference test a laboratory test albumin-creatinine ratio-ACR (let’s call *RT*_*0*_ the class of such reference tests).

They take into account ten studies in their article. Consider for example Lloyd et al. [22], which measures the accuracy of semiquantitative Clinitek® microalbumin urine dipstick with a cutoff value indicating albumineria at 3.4 mg/mmol (let’s call *IT*_*1*_ the class of such index tests), with a laboratory ACR test with the same cutoff value as a reference (let’s call *RT*_*1*_ the class of such reference tests). A sample **s**_**1**_ of 204 diabetic patients (labelled here **p**_**1,1**_, **p**_**1,2**_,…, **p**_**1,204**_) was considered. On each of those patients, one measurement of *IT*_*1*_ called **a**_**1,i,1**_ and one of *RT*_*1*_ called **rt**_**1,i,1**_ is performed. The 2x204 = 408 processual entities are all part of a general tests execution process labelled **tests_execution**_**s1,***IT1,RT1*_, which leads after computation to the informational entity **estimate** ^**Se**^_**1**_, giving the proportion of measure pairs in which *IT*_*1*_ led to a positive result among those in which *RT*_*1*_ led to a positive result. This proportion is 83.8 %, and therefore, the value f4(**s**_**1**_,*IT*_*1*_,*RT*_*1*_) of the informational entity **estimate** ^**Se**^_**1**_ is 0.838.

Writing **g** the human population, we have **s**_**1**_ **part_of g**; also, *RT*_*1*_ *is_a RT*_*0*_ and *IT*_*1*_ *is_a IT*_*0*_ *.* Therefore, f_4_(**s**_**1**_,*IT*_*1*_,*RT*_*1*_) provides an estimate of f_2_(**g**,*IT*_*0*_,*RT*_*0*_), which is the sensitivity value of a point-of-care test in detecting albuminuria in the general population. However, other studies are pooled with this one by McTaggart and colleagues [8] to provide a better estimate of f_2_(**g**,*IT*_*0*_,*RT*_*0*_). All together, they lead to the value h(**s**_**1**_ *,IT*_*1*_,*RT1*,…,**s**_**10**_,*IT*_*10*_,*RT*_*10*_) which provides an estimate of the value of f_2_(**g**,*IT*_*0*_,*RT*_*0*_).

Note that the ten studies taken into account in this meta-analysis include different kinds of patients. Seven studies involve each a different sample of patients (let’s call them **s**_**1**_, **s**_**2**_, …., **s**_**7**_) with diabetes mellitus, one of them (**s**_**7**_) involving young patients with type 1 diabetes. Two studies consider samples of patients (**s**_**8**_ and **s**_**9**_) with kidney disease, diabetes mellitus, or both. Finally, one study includes a sample (**s**_**10**_) of patients treated for advanced chronic kidney disease in a renal outpatient clinic. Let’s call **g** the human population, **g**_**1**_ the members of g who have diabetes mellitus, **g**_**2**_ the members of **g** who have a kidney disease and **g**_**0**_ the members of **g** who have either diabetes mellitus or a kidney disease (that is, **g**_**0**_ is the mereological sum of **g**_**1**_ and **g**_**2**_). All **s**_**i**_ are part of **g**, the human population. Thus, the meta-analysis made by McTaggart and colleagues [8] provides an estimation of f_2_(**g**,*IT*_*0*_,*RT*_*0*_) or f_2_(**g**_**0**_,*IT*_*0*_,*RT*_*0*_). If the meta-analysis had been performed on **s**_**1**_ **-s**_**7**_ only, then it would have provided an estimation of f_2_(**g**_**1**_,*IT*_*0*_,*RT*_*0*_); and if it had been performed on samples of patients with kidney disease only, then it would have provided an estimation of f_2_(**g**_**2**_,*IT*_*0*_,*RT*_*0*_).

Note also that various cutoff values can be used to define the presence of albuminuria, varying between 2.65 mg/mmol to 3.4 mg/mmol, and those values are chosen by the medical sub-community who is conducting the study (the same cutoff value is taken for both *IT*_*0*_ and *RT*_*0*_ in each study). Therefore, the classes *IT*_*0*_ and *RT*_*0*_, which mention ‘detecting albuminuria’ without specifying a cutoff value, are not scientifically defined: those classes are not universals, but rather collection of particulars [19] whose nature is partly social ([8] acknowledge this limitation in their meta-analysis).

Alternative meta-analysis could use a subset of those studies to estimate various sensitivities, for example the sensitivity f_2_(**g**_**1**_,*IT*_*1*_,*RT*_*1*_) of point-of-care test with a reference of laboratory ACR test, with albuminuria defined as ACR greater than 3.4 mg/mmol, in the reference class of patients with diabetes mellitus; or the sensitivity f_2_(**g**_**2**_,*IT*_*2*_,*RT*_*2*_) of point-of-care test, with a reference of laboratory ACR test, with albuminuria defined as ACR greater than 2.65 mg/mmol, in the reference class of patients with kidney disease; etc. A well-founded semantic representation of sensitivity should thus make clear what is the reference class, as well as the class of index test and reference test.

## Discussion and conclusions

We have thus provided a practically tractable formalization of IPs in a realist ontology, which clearly dissociates IPs’ real values, their estimates and the related proportion measurements. It has defined the central entities that are concerned by an IP estimation in a way that is compliant with OBO Foundry. In particular, it addresses the difficulty of considering possible, non-actual conditions in a realist ontology based on BFO by introducing dispositions.

This model could then be extended in three directions. A first step would be to clarify the ontological status of the two following entities: sample sizes on one hand; and 95 % confidence interval for sensitivity and specificity values on the other hand. A second step would be to clarify the relations *se_of_test* and *se_for_disease*, which could be reduced to basic relations and entities already accepted in the OBO Foundry. A third step would be to use this model in an ontology-based diagnostic system that would compute positive predictive values or negative predictive values from the prevalence, sensitivity and specificity values. More generally, it could be articulated with medical Bayesian networks. As a matter of fact, the notion of medical test used here could be generalized to a very general notion of test consisting in inferring the presence of an entity on the basis of the knowledge of the presence of another entity; as such, it could serve as a foundation for the integration of Bayesian reasoning into ontologies.

This model could be used in two kinds of computer applications targeted at two different kinds of audiences. First, clinicians could determine more easily which kind of sensitivity and specificity (or PPV and NPV) estimates they could use when diagnosing a disease for a given patient, by having a clearer view of the subjects’ characteristics in each samples on which those IP estimates are based. As a matter of fact, section 3.4 illustrates how an ontological analysis can make explicit what are the index test, the reference test and the sample associated with a sensitivity estimation. Universal qualities that are instantiated by all members of the sample - such as having diabetes mellitus, being a man, being more than 65 years old, etc. - would enable to determine what could be the reference class **g** associated with a sensitivity estimate. This enables to determine, when applying some given IP values to a specific patient with given characteristics, whether this application is warranted or not.

Second, statisticians could determine more easily which kind of sensitivity estimates they could aggregate together. If several estimations of IPs are represented ontologically according to the structure shown above, one could use this ontological structure to determine which estimations of IPs could be combined to obtain a finer estimate. First, one would have to find a group **g**_**0**_ that would encompass the reference classes (such as **g**_**1**_ and **g**_**2**_) associated with those studies. Second, one would have to analyze whether there exists some general index test class such as *IT*_*0*_ (resp. some general reference test class such as *RT*_*0*_) which would subsume the various index tests classes such as *IT*_*1*_ and *IT*_*2*_ (resp. reference tests such as *RT*_*1*_ and *RT*_*2*_) that are used in those studies. Once those are found, one could use meta-analytic methods to derive a value for f_2_(**g**_**0**_,*IT*_*0*_,*RT*_*0*_) from the other studies. Future work will aim at building an ontology of medical tests to facilitate finding such encompassing index and reference test classes.

As it takes into account the dependence of IPs upon the group of people considered, it has the potential to contribute to the development of precision medicine [23] in context of learning health systems [24, 25], an emerging approach that takes into consideration patients characteristics and dispositions, including individual variability in genes, to offer more personalized preventive, diagnostic and therapeutic strategies.

## General abbreviations for indicators of performance

IP: (Bayesian) Indicators of performance
NPV: Negative predictive value
PPV: Positive predictive value
Prev: Prevalence
Se: Sensitivity
Sp: Specificity

## Other general abbreviations

ACR: Albumin-creatinine ratio
RF: Rheumatoid factor

## Classes and instances abbreviations for disposition-related entities

**d**^Prev^_**g**,*M*_: Disposition (borne by the group **g**) that a person randomly drawn among the individuals in **g** would have *M*
**d**^Se^_**g**,*IT,M*_: Disposition (borne by the group **g**) that a person randomly drawn among the individuals of **g** who have *M* would have a positive result to *IT*; this is an instance of *D* ^*Se*^_*IT,M*_
*D*^*Se*^_*IT,M*_: A subclass of *Sensitivity disposition* such that *D* ^*Se*^_*IT,M*_ *se_for_disease M* and *D* ^*Se*^_*IT,M*_ *se_of_test IT*
*D*^*Sp*^_*IT,M*_: A subclass of *Specificity disposition* such that *D* ^*Sp*^_*IT,M*_ *sp_for_disease M* and *D* ^*Sp*^_*IT,M*_ *sp_of_test IT*
*T*_**g**_: The process of drawing randomly a person in **g**; the triggers of **d** ^Prev^_**g**,*M*_ are instances of *T*_*g*_
*R*_**g**,M_: The process of drawing by *T*_**g**_ someone who has *M*; the realizations of **d** ^Prev^_**g**,*M*_ are instances of *R*_**g***,M*_
*R*^Se^_**g***,IT,M*_: The process of drawing by *T* ^Se^_**g***,IT,M*_ someone who got a positive result to *IT*; the realizations of **d** ^Se^_**g**,*IT,M*_ are instances of *R* ^Se^_**g***,IT,M*_
*T*^Se^_**g***,IT,M*_: The process of performing test *IT* on the individuals in **g**, and then drawing randomly an individual among those who have the disease *M*; the triggers of **d** ^Se^_**g**,*IT,M*_ are instances of *T* ^Se^_**g***,IT,M*_

## Other classes abbreviations

*IT / IT*_*0*_*/ IT*_*1*_*/ IT*_*2*_: A subclass of *Medical test* which is an index test (test whose indicator of performance is being estimated)
*M*: A subclass of *Disease*
*RT / RT*_*0*_*/ RT*_*1*_*/ RT*_*2*_: A subclass of *Medical test* which ﻿is a reference test

## Other instances abbreviations

**g** / **g**_**0**_ / **g**_**1**_ / **g**_**2**_: An instance of *Collection of humans* which is a general human population
**p**_**i**_**/ q**_**j**_: An instance of *Human*
**it**_**1,i**_ (resp. **it**_**2,j**_**)**: An instance of (index) *Medical test* performed on person **p**_**i**_ (resp. **q**_**j**_)
**rt**_**1,i**_ (resp. **rt**_**2,j**_): An instance of (reference) *Medical test* performed on person **p**_**i**_ (resp. **q**_**j**_)
**s** / **s**_**1**_ / **s**_**2**_: An instance of *Sample of humans*

## Functions abbreviations

f_1_(*IT,M*): Proportion of individuals who get a positive result to *IT*, among individuals who have *M*
f_2_(**g**,*IT,M*): Proportion, among members of **g** who have *M*, of those who would get a positive result to *IT if the test IT was realized on them*; this is the real sensitivity value of *IT* for *M*
f_3_(**g**,*IT,RT*): Proportion, among members of **g** who would get a positive result to *RT if the test RT was realized on them*, of those who would get a positive result to *IT if the test IT was realized on them*
f_4_(**s**,*IT,RT*): Proportion, among members of sample **s** who had a positive result to *RT*, of those who got a positive result to *IT;* this is an estimate of the real sensitivity value of *IT* for *M*, performed on a sample **s**, with *RT* as a reference test
f’_2_(**g**,*IT,M*): Proportion, among members of **g** who don’t have *M*, of those who would get a negative result to *IT if the test IT was realized on them*; this is the real specificity value of *IT* for *M*
f’_4_(**s**,*IT,RT*): Proportion, among members of sample **s** who had a negative result to *RT*, of those who got a negative result to *IT;* this is an estimate of the real specificity value of *IT* for *M*, performed on a sample **s**, with *RT* as a reference test
h(**s**_**1,**_*IT*_*1*_*,RT*_*1*_,**s**_**2**_,*IT*_*2*_*,RT*_*2*_): Estimate of the sensitivity value obtained by aggregating the estimate on sample **s**_**1**_ and the estimate on sample **s**_**2**_

## Footnotes

1 These will be abbreviated in the following as “a test *IT*” and “the patient has *M*”. Note that a test may aim at diagnosing a disease, in which case it can be called “indicator of diagnostic performance”. However, it may also aim at evaluating the presence of a disorder, a pathological process [26], a predisposition to a disease, a sign, a symptom, or other various medically relevant entities (such as a glycemia higher than 1.26 g/l). Several tests results can then be considered to draw a diagnostic conclusion for a disease. Therefore, in the general case, indicators of performance are indicators of *assay* performance rather than indicators of *diagnostic* performance (we thank an anonymous reviewer for this suggestion of terminology). Also, a test does not need to be performed on a human – it can be performed on a non-human animal. In the following, we will consider tests aiming at diagnosing a disease on a human, but our considerations can be straightforwardly adapted to tests aiming at evaluating another medically relevant entity on a human or non-human animal.

2 In practice, such a test is not perfect; thus, it could be analyzed as a chain of two tests: one that detects the rheumatoid factor on the basis of e.g., some chemical reaction, and another one that detects rheumatoid arthritis on the basis of the presence of the rheumatoid factor.

3 More specifically, it should be interpreted as the expected value of such a proportion – but we will ignore here this additional subtlety.

4 The article will concentrate on the case of sensitivity, but it can be similarly adapted to other IPs.

5 Here again (see footnote 3), this should be interpreted as the expected value of such a proportion.

6 At least for all practical purposes: from a theoretical point of view, every measurement can be wrong, even pure observations.

7 If one assumes that the sample is representative of the target population, there should be no selection bias (which occurs when proper randomization is not achieved). However, the sensitivity values that would be obtained using two different samples could be slightly different since randomness at the selection process will yield slightly different samples. That is why statisticians use confidence interval for characterizing sensitivity and specificity.

8 We might also speak of a “sensitivity in a sample” for the function f_2_(**s**,*IT,M*), that is, the proportion of people who are tested positive by *IT* among the diseased person in the sample **s**. But it might be confusing to speak of both the “sensitivity in a target population” and the “sensitivity in a sample”; and the first and the second arguments above may justify keeping the label “sensitivity” for this proportion in a target population **g** – that is, for f_2_(**g**,*IT,M*).

9 Let us summarize. On one hand, f_2_(**g**,*IT,M*) is the value of a non-actual proportion (because the test *IT* is not performed on all members of **g**), which cannot be known with certainty, but only estimated. On the other hand, both f_4_(**s**,*IT,RT*) and f_2_(**s**,*IT,M*) (see footnote 8) are values of actual proportions (because the tests *IT* and *RT* are performed on all members of **s**); and although f_2_(**s**,*IT,M*) cannot be known with certainty (because we cannot know with certainty who has the disease: we can only use a reference test – at best the gold standard – to determine who are those individuals), f_4_(**s**,*IT,RT*) can be known with certainty for all practical purposes (because we can know with certainty who got a positive result to *RT*).

10 We have created an ontology according the lines of what is described below, built on OBI, called BIPO (Bayesian Indicator of Performance Ontology). It can be found at https://github.com/OpenLHS/BIPO. It contains 24 classes, 12 object properties, 2 data properties and 42 logical axioms.

11 We will not take a stance on whether *Medical_test* should be interpreted as identical to OBI:*Assay*, as proposed by [27].

12 Note that in some cases, several pairs of tests will be performed on a person. See e.g., Kimberger et al. (2007), which measures the accuracy of a temporal artery thermometer in detecting fever (defined as a temperature greater than 37.8 °C), with respect to a reference standard given by a bladder thermometer: four measurement pairs of temporal artery temperature and bladder temperature are performed on each of the seventy patients of the sample considered by the authors. To represent such a case, one can introduce for every human **p**_**i**_ a sequence of four reference tests **rt**_1,i,1_, **rt**_1,i,2_, **rt**_1,i,3_ and **rt**_1,i,4_ .and four index tests **it**_1,i,1_, **it**_1,i,2_, **it**_1,i,3_ and **it**_1,i,4_; but the formalization that is described below remains similar.

13 See e.g., http://vassarstats.net/clin1.html for an example of webpage supporting this kind of computation.

14 As a reminder, not only the values of PPV and NPV but also the values of sensitivity and specificity depend on the group under consideration (this is the spectrum effect), and it is not the task of the ontologist to determine which ones should be idealized as constant ﻿(﻿for all practical matters) across groups and which ones should be considered as variable: the task of the ontologist is to represent those values and the entities those values depend upon.

15 [15] assigned a probability to a triplet (**d**,*T*,*R*) rather than to a disposition **d**, because it had to take into account dispositions that may have several classes of triggers or realizations (that is, multi-trigger and multi-track dispositions [20]). However, in the present situation, **d** ^*Se*^_**g***,M*_ is simple-trigger and simple-track: all its triggers are instances of *T* ^*Se*^_**g**_ , and all its realizations are instances of *R* ^*Se*^_**g**,*M*_. Therefore, the probability value assigned to (*d* ^*Se*^_**g***,M*_,*T* ^*Se*^_**g**_ *,R* ^*Se*^_**g***,M*_) can be, for practical matters, assigned directly to *d* ^*Se*^_**g***,M*_.

16 Such dispositions should not be confused with other dispositions in the medical domain. First, diseases have been formalized as dispositions by the Ontology for General Medical Sciences (OGMS) [26]. Second, there can be predispositions to diseases that could be formalized as disposition. However, the disposition to draw randomly, among the individuals of ***g*** who have *M*, someone who is tested positive by *IT*, exists independently of whether the disease (or a predisposition to this disease) is formalized or not as a disposition. Note also that this disposition inheres in a group of people, whereas a disease as a disposition (as formalized by OGMS), or a predisposition to a disease, inheres in a single person.

17 In general, we cannot determine in practice with certainty which individuals of *g* have *M*, and which do not (see the discussion about gold standard tests above); but the practical impossibility to realize this trigger does not preclude to define this entity.

18 We could also introduce the entity **real_sensitivity**_**g***,IT,M*_ instance of *Data_item*, as a sibling of **estimate** ^*Se*^_1_ such that **real_sensitivity**_**g,***IT,M*_ **has_specified_value** f_2_(**g** *,IT,M*) (cf. [14], in which **real_sensitivity**_**g,***IT,M*_ was denoted **se**_**g,***IT,M*_). However, the value f_2_(**g** *,IT,M*) assigned to such an entity will never be known with certainty. We could substitute to this value the best estimate of the sensitivity value, as was proposed in [14]; however, such a model could not represent in a single ontology various estimates of the same sensitivity – whereas it is possible in the present framework, which also makes unnecessary the introduction of the informational entity **real_sensitivity**_***g****,IT,M*_.

19 It is important to differentiate what a sensitivity estimate is about (namely a disposition) from how it has been mathematically obtained (for example, by weighting different proportion measurements) – as explained earlier, the latter will not be represented in the ontology, as various mathematical methods can be used.

## References

[1] Ledley RS, Lusted LB (1959). Reasoning foundations of medical diagnosis. Science.

[2] Peacock J, Peacock P (2011). Oxford Handbook of Medical Statistics.

[3] Brenner H, Gefeller O (1997). Variation of sensitivity, specificity, likelihood ratios and predictive values with disease prevalence. Stat Med.

[4] Park HB, Yokota A, Gill HS, El Rassi G, McFarland EG (2005). Diagnostic accuracy of clinical tests for the different degrees of subacromial impingement syndrome. J Bone Joint Surg Am..

[5] Hanson V, Rexler ED, Kornreich H (1969). The relationship of rheumatoid factor to age of onset in Juvenile rheumatoid arthritis. Arthritis Rheum.

[6] Moons KGM, van Es G-A, Deckers JW, Habbema JDF, Grobbee DE (1997). Limitations of sensitivity, specificity, likelihood ratio, and bayes’ theorem in assessing diagnostic probabilities: a clinical example. Epidemiology.

[7] Niven DJ, Gaudet JE, Laupland KB, Mrklas KJ, Roberts DJ, Stelfox HT (2015). Accuracy of peripheral thermometers for estimating temperature: a systematic review and meta-analysis. Ann Intern Med.

[8] McTaggart MP, Newall RG, Hirst JA, Bankhead CR, Lamb EJ, Roberts NW, Price CP (2014). Diagnostic accuracy of point-of-care tests for detecting albuminuria: a systematic review and meta-analysis. Ann Intern Med.

[9] Reilly P, Macleod I, Macfarlane R, Windley J, Emery R (2006). Dead men and radiologists don’t lie: a review of cadaveric and radiological studies of rotator cuff tear prevalence. Ann R Coll Surg Engl..

[10] Smith B, Ashburner M, Rosse C, Bard J, Bug W, Ceusters W, Goldberg LJ, Eilbeck K, Ireland A, Mungall CJ (2007). The OBO Foundry: coordinated evolution of ontologies to support biomedical data integration. Nat Biotechnol.

[11] Grenon P, Smith B, Goldberg L, Pisanelli D (2004). Biodynamic ontology: applying BFO in the biomedical domain. Ontologies in medicine.

[12] Ceusters W (2012). An information artifact ontology perspective on data collections and associated representational artifacts. Stud Health Technol Inform.

[13] da Costa PCG, Laskey KB, Laskey KJ, Costa PCG, d'Amato C, Fanizzi N, Laskey KB, Laskey KJ, Nickles M, Pool M (2008). PR-OWL: A Bayesian Ontology Language for the Semantic Web. Uncertainty Reasoning for the Semantic Web I.

[14] Soldatova LN, Rzhetsky A, De Grave K, King RD (2013). Representation of probabilistic scientific knowledge. J Biomed Semant.

[15] Barton A, Duvauferrier R, Burgun A. Formalization of indicators of di agnostic performance in a realist ontology. In: Couto F M, Hastings J, editors. Proceedings of 6th International Conference on Biomedical Ontology (ICBO2015). CEUR Workshop Proceedings 1515, CEUR-WS.org; 2015. p. 63-70.

[16] Zheng J, Harris MR, Masci AM, Lin Y, Hero A, Smith B, He Y, Hogan W, Arabandi S, Brochhausen M (2014). OBCS: The Ontology of Biological and Clinical Statistics. Proceedings of the 5th International Conference on Biomedical Ontology.

[17] Brinkman RR, Courtot M, Derom D, Fostel JM, He Y, Lord P, Malone J, Parkinson H, Peters B, Rocca-Serra P, Ruttenberg A, Sansone S-A, Soldatova LN, Stoeckert CJ, Turner JA, Zheng J, OBI consortium (2010). Modeling biomedical experimental processes with OBI. J Biomed Semant.

[18] Jansen L, Schulz S (2011). Grains, components and mixtures in biomedical ontologies. J Biomed Semant.

[19] Smith B, Ceusters W (2010). Ontological realism: A methodology for coordinated evolution of scientific ontologies. Appl Ontol.

[20] Röhl J, Jansen L (2011). Representing dispositions. J Biomed Semant.

[21] Schulz S, Martínez-Costa C, Karlsson D, Cornet R, Brochhausen M, Rector A, Garbacz P, Kutz O (2014). An Ontological Analysis of Reference in Health Record Statements. Proceedings of the 8th International Conference on Formal Ontology in Information Systems (FOIS2014).

[22] Lloyd, Mariana M, Johannes K, H. Van Jaarsveld. Evaluation of point-of-care tests for detecting microalbuminuria in diabetic patients. South African Family Practice 53.3. 2011;281–286.

[23] Mirnezami R, Nicholson J, Darzi A (2012). Preparing for precision medicine. N Engl J Med..

[24] Delaney BC, Curcin V, Andreasson A, Arvanitis TN, Bastiaens H, Corrigan D, Ethier J-F, Kostopoulou O, Kuchinke W, McGilchrist M, Van Royen P, Wagner P (2015). Translational medicine and patient safety in Europe: TRANSFoRm—architecture for the Learning Health System in Europe. BioMed Res Int.

[25] Friedman C, Rubin J, Brown J, Buntin M, Corn M, Etheredge L, Gunter C, Musen M, Platt R, Stead W, Sullivan K, Van Houweling D. Toward a science of learning systems: a research agenda for the high-functioning Learning Health System. J Am Med Inform Assoc. 2014;22(1):43-50. 10.1136/amiajnl-2014-002977 PMC4433378 25342177

[26] Scheuermann RH, Ceusters W, Smith B (2009). Toward an ontological treatment of disease and diagnosis.

[27] Jensen M, Cox AP, Bona JP, Duncan W, Ray PL, Diehl AD, Hogan W, Arabandi S, Brochhausen M (2014). Applications of OBI “assay.”. Proceedings of the 5th International Conference on Biomedical Ontology.

